# Acceptability and perception of COVID-19 vaccines among foreign medical students in China: A cross-sectional study

**DOI:** 10.3389/fpubh.2023.1112789

**Published:** 2023-03-28

**Authors:** Clement Arthur, Zhen Dong, Hermas Abudu, MengLu Li, George N. Chidimbah Munthali, Chunming Zhang, Sen Zhang, Rui Han, Stephen Ogbordjor, Amos Dormocara, Lina Ja, Di Zhang, Haili Zhang, Hui Huangfu

**Affiliations:** ^1^Department of Otolaryngology Head and Neck Surgery, First Hospital of Shanxi Medical University, Taiyuan, Shanxi, China; ^2^Shanxi Key Laboratory of Otorhinolaryngology Head and Neck Cancer, First Hospital of Shanxi Medical University, Taiyuan, Shanxi, China; ^3^Department of Pharmaceutical Sciences, Soochow University, Suzhou, Jiangsu, China; ^4^Department of Otolaryngology, Regional Hospital Sunyani, Sunyani, Ghana; ^5^College of Overseas Education Chengdu University, Chengdu, Sichuan, China; ^6^Department of Finance and Accounting, Mzuzu University, Mzuzu, Malawi; ^7^First Klass Klassic Hospital, Konongo, Ghana; ^8^Soochow University Department of Pharmaceutical Sciences Pharmaceutics, Suzhou, Jiangsu, China; ^9^Department of Basic Medical, Shanxi Medical University, Jinzhong, Shanxi, China

**Keywords:** COVID-19, COVID-19 vaccine, international students, medical students, pandemic

## Abstract

**Background:**

Acceptability and perception of the COVID-19 vaccine among different social groups have been the subject of several studies. However, little is known about foreign medical students in Chinese universities.

**Aim:**

This study, therefore, fills the literature gap using a focus group technique to assess the acceptance and perception of the COVID-19 vaccine among foreign medical students in China.

**Methods:**

The study adopted an online cross-sectional survey method following the Chinese universities' lockdowns to collect the data between March and April 2022. A data collection questionnaire was developed, and then the link was shared with the respondents through key informants in different universities in China to obtain the data. The data collection process only included foreign medical students who were in China from May 2021 to April 2022. The authors received a total of 403 responses from the respondents. During data processing, we excluded 17 respondents since they were not in China while administering the questionnaire to enhance the data validity. The authors then coded the remaining 386 respondents for the estimation process. We finally applied the multilinear logistics regression technique to model the COVID-19 vaccine acceptance with the response or influencing factors, including the mediating factors among the foreign medical students in China.

**Results:**

The data statistics show that 4.9% of the respondents were younger than 20 years, 91.5% were 20–40 years old, and 3.6% were older than 40 years; 36.3% of respondents were female subjects and 63.7% were male subjects. The results also show that the respondents are from six continents, including the African continent, 72.4%, Asia 17.4%, 3.1% from Europe, 2.8% from North America, 1.6% from Australia, and 2.3% from South America. The mediation analysis for the gender variable (β = 0.235, *p* = 0.002) suggests that gender is a significant channel in COVID-19 vaccine acceptance and perception among foreign medical students in China. Also, the main analysis shows that opinion on the safety of the vaccine (β = 0.081, *p* = 0.043), doses of the vaccine to receive (β = 0.175, *p* = 0.001), vaccine safety with some side effects (β = 0.15, *p* = 0.000), and the possibility of acquiring COVID-19 after vaccination (β = 0.062, *p* = 0.040) are all positive factors influencing vaccine acceptability and perception. Also, the home continent (β = −0.062, *p* = 0.071) is a negative factor influencing COVID-19 vaccine acceptance and perception. Furthermore, the finding shows that fear perceptions has affected 200 (51.81%) respondents. The medical students feared that the vaccines might result in future implications such as infertility, impotence, and systemic health conditions such as cardiovascular, respiratory, or deep vein thrombosis. In addition, 186 (48.19%) students feared that the vaccines were intended to shorten life expectancy.

**Conclusion:**

COVID-19 vaccination acceptability and perception among medical students in China is high, most predominantly due to their knowledge of medicine composition formulation. Despite widespread acceptance by the general public and private stakeholders, we concluded that vaccination resistance remains a significant factor among medical students and trainees. The study further adds that in considering the COVID-19 vaccine, the factor of the home continent plays a significant role in vaccine hesitancy among foreign medical students. Also, knowledge, information, and education are important pillars confronting new medicine administered among medical trainees. Finally, there is a low rate of COVID-19 vaccine hesitancy among foreign medical students in China. The study, therefore, recommends targeted policy strategies, including sensitization, detailed public information, and education, especially for medical colleges and institutions on the COVID-19 vaccination, to achieve 100%. Furthermore, the study recommends that future researchers explore other factors influencing accurate information and education for successful COVID-19 vaccination implementation.

## Introduction

Coronavirus infectious disease 2019 (COVID-19) was declared a pandemic in March 2021 by the World Health Organization (WHO) (1, 2). The literature suggests that COVID-19 causes severe acute respiratory syndrome coronavirus (SARS-CoV-2) and continues to impact global public health significantly (3, 4). Many efforts by governments to combat the disease have led to implementing health prevention policies, including lockdowns, social distancing in public places, hand hygiene, using masks, and finally, the rollout of vaccines (2) as a last resort in health management protocols.

There are many COVID-19 vaccines available in the world market, specially manufactured by world high medical suppliers in countries like the United States of America, the United Kingdom, China, Russia, and India (5). Despite some of the challenges, usually encountered such as trust issues, the WHO has approved all COVID-19 vaccines after careful checks of the trial testing procedures, with special attention to ensuring human life safety (6). The approval process has led to a high level of trust, and not surprisingly, there has been a dependence on the COVID-19 vaccine, just like in previous epidemics management measures. Many developed countries depend on their vaccines while developing countries appear to have more trust in imported vaccines from developed countries because they cannot manufacture their own (6) due to limited medical resources and capacity. Because of mistrust in the sources of vaccines, the acceptance and perception of COVID-19 vaccines among people are affected due to religious beliefs and cultural and personal reasons (7). In some parts of the world, the vaccine has been received with high acceptability due to the system of governance and fear of the impact of COVID-19 (8). According to the literature, vaccine resistance was independently predicted as ignorance of vaccination eligibility, worry about vaccine side effects and effectiveness, and mistrust of the government (9). Greater awareness of the risk associated with COVID-19 appeared to lessen vaccine hesitancy (9). Several people have raised concerns about the scarcity of vaccination-related information as a prerequisite in vaccine introduction before the publication of safety and efficacy data (10, 11). Although vaccine reluctance has declined over time, health education programs designed to increase vaccination awareness and promote public confidence in government institutions would be beneficial (9). Enhancing health promotion and lowering obstacles to COVID-19 vaccination is crucial (10–12). Similarly, many participants (10–12) lacked knowledge of the COVID-19 vaccinations, although health authorities approved vaccine use in pregnant women and children over 12 years of age. Health knowledge updates, such as vaccinations for expectant mothers and children, should be provided as ongoing awareness campaigns that target all demographic groups (13). The COVID-19 mRNA vaccines are well known, but the confidence level of successful COVID-19 vaccine manufacturers differ significantly from one another (12). The pandemic may be a good opportunity to raise public understanding of vaccines, highlighting the importance of effective and ongoing scientific communication in the battle against the disease (13).

The literature studies show that the reluctance to vaccine acceptance is one of the biggest obstacles to conducting successful immunization programs (14) in many parts of the world. Studies show that adopting any vaccination among healthcare professionals is one important way to improve its acceptance efficacy and safety (15). Also, social media is yet another important key player in influencing the attitude toward the acceptance of COVID-19 vaccines (16). Furthermore, factors such as profession, alcohol intake, and knowledge and attitude toward the COVID-19 vaccination impact the intention to receive the vaccine (17). A study shows that Indian college students have a highly positive intention of receiving COVID-19 vaccines, although one-third were unsure or hesitant to receive vaccines (18). It is essential to relieve peoples' anxiety and improve their confidence through health education (19). Furthermore, studies suggest that gender (male) with higher educational status, urban housing, and using television or radio have COVID-19 information sources that are strongly associated with research participants' knowledge levels (20). The activities of the government, particularly through social distance measures, have favorably increased the feelings of safety and security among overseas students (21). The social media platform has significantly contributed to the spread of information and has praised Chinese institutions for its ongoing COVID-19 alerts that enabled students to have a complete understanding (22). Since the media and other related parties play a significant role in the acceptance or otherwise of the vaccine, the governments have advised them to spread accurate, reliable, and consistent COVID-19 vaccine information to foster public trust (23).

Studies show that the positive effects of COVID-19 vaccination are evident in many countries, but certain sectors still deny its impact (24). Governments have used various COVID-19 vaccination implementation strategies based on national policies (25). A large body of research exists about people's perceptions and attitudes toward COVID-19 vaccines among diverse social categories of society (10–12). Studies suggest that some social groupings still have a larger knowledge gap and unfavorable views on the subject (26). In line with the literature, it is important to examine the COVID-19 vaccine acceptance and perceptions among different groupings (27). According to studies, public health efforts have stressed the alleged advantages of vaccinations and the anticipated risks of skipping; however, the majority of students had weak knowledge and favorable perceptions (28). The WHO Strategic Advisory Group of Experts (29) described vaccine hesitancy as a delay in acceptance or refusal of immunization despite the availability of vaccination services. Studies including (30) COVID-19 vaccine hesitancy is substantially high, between 10% and 37% among medical students. As a result of the high hesitancy among medical students, it is an important subject, and currently, there needs to be more studies among foreign medical students in Chinese universities (31). The results could be instructive for decision-makers as they try to maximize the spread of vaccines (32). Therefore, this study seeks to contribute to the literature debate in the context of foreign medical students in Chinese universities. The relevance of the survey among foreign medical students' acceptance or otherwise of the COVID-19 vaccine may play a significant role in the general international students since they have adequate knowledge and information on the vaccine composition matrix in China. This may also be relevant to the WHO-SAGE method, which states that countries should use the Convenient, Complacency, and Confident (3-Cs) (33) model in the COVID-19 vaccine administration worldwide. The 3-Cs approach seeks to boost and break barriers to accepting the COVID-19 vaccine across all social groupings, such as foreign medical students (34, 35). So, the relevance of the study further seeks to assess the foreign medical students' trust in getting vaccinations in terms of no need to pay for vaccines, high vaccine availability, no language barriers, and availability of information on the vaccine to reduce any complacency among social categories such as students. In this regard, China is among the few countries to use the COVID-19 administering challenges to implement technological innovations to help build on their systems, particularly when students return to school after the lockdown periods. Therefore, this study has academic and policy relevance in broadening the understanding of the COVID-19 vaccine acceptance and assessing the efficacy of the 3-Cs model among foreign medical students on vaccine hesitancy in China (36). This study aims to model and evaluate the influencing factors (i.e., attitude, beliefs, experiences, reactions, and feelings) affecting COVID-19 vaccination acceptance among international medical students in Chinese universities and Colleges.

## Materials and methods

The authors adopted the STROBE guideline in this study starting with the title (item 1) till funding (item 22) (34). In this section, we applied items 4–12 in guiding the cross-sectional study consistent with the STROBE checklist, see Appendix A. The authors adopted the focus group technique to draw upon the foreign medical students' attitudes, beliefs, experiences, reactions, and feelings through an online data-collecting survey which would not be feasible using other methods such as observational and one-on-one interviewing techniques because of COVID-19 lockdown protocols.

### Population, sample size, and technique

In this study, we targeted all levels of international foreign medical students enrolled in Chinese universities, including undergraduate, graduate, and postgraduate. Therefore, we used a standard sampling technique to source the data from the students in different universities using an online survey technique. Consistent with the literature, we used a data sampling technique developed by Yamane [8/11] to generate the sample size. Consistent with [8/11], we used Yamane (1967:886)^32^ to calculate a sample size with a 95% confidence level and *P* = 0.05^32^. That is, according to Yamane, the sample size is (n) =N/(1+N(e)^2^), where N is the population size and e is the level of precision. In applying this formula, the authors estimated the total number of foreign medical students in Chinese universities to be over 10,000^1^ population. Therefore, the authors used the estimated 10,700 foreign medical students in China as a base using the Yamane formula to calculate the sample size of 386.

### Study and questionnaire design

In this study, we used a qualitative research design using a one-time data collection technique involving a cross-sectional survey conducted from March to April 2022 to source data from international foreign medical students in Chinese universities. The authors designed the questionnaire in two sections. The first part of the questionnaire captures data on social demographic characteristics such as gender, age, program major, years in China, and religion. The second part of the questionnaire captured data on COVID-19 vaccination acceptability and perceptions formulated based on the literature (18).

### Data collection and respondents recruitment procedures

First and foremost, the authors identified key informants (leaders) across the targeted Chinese universities in February 2022. So, the key informants or cadets connected us to foreign medical students. Furthermore, the authors sent the self-administrated online survey questionnaire link shared on the media, including WeChat and WhatsApp, and university groups in China through the identified key informants. The online questionnaire technique allowed the students to self-administer the online survey link shared, and responses were automatically generated after completing the questionnaire.

### Respondents' inclusion and exclusion criteria

**Figure 1:**
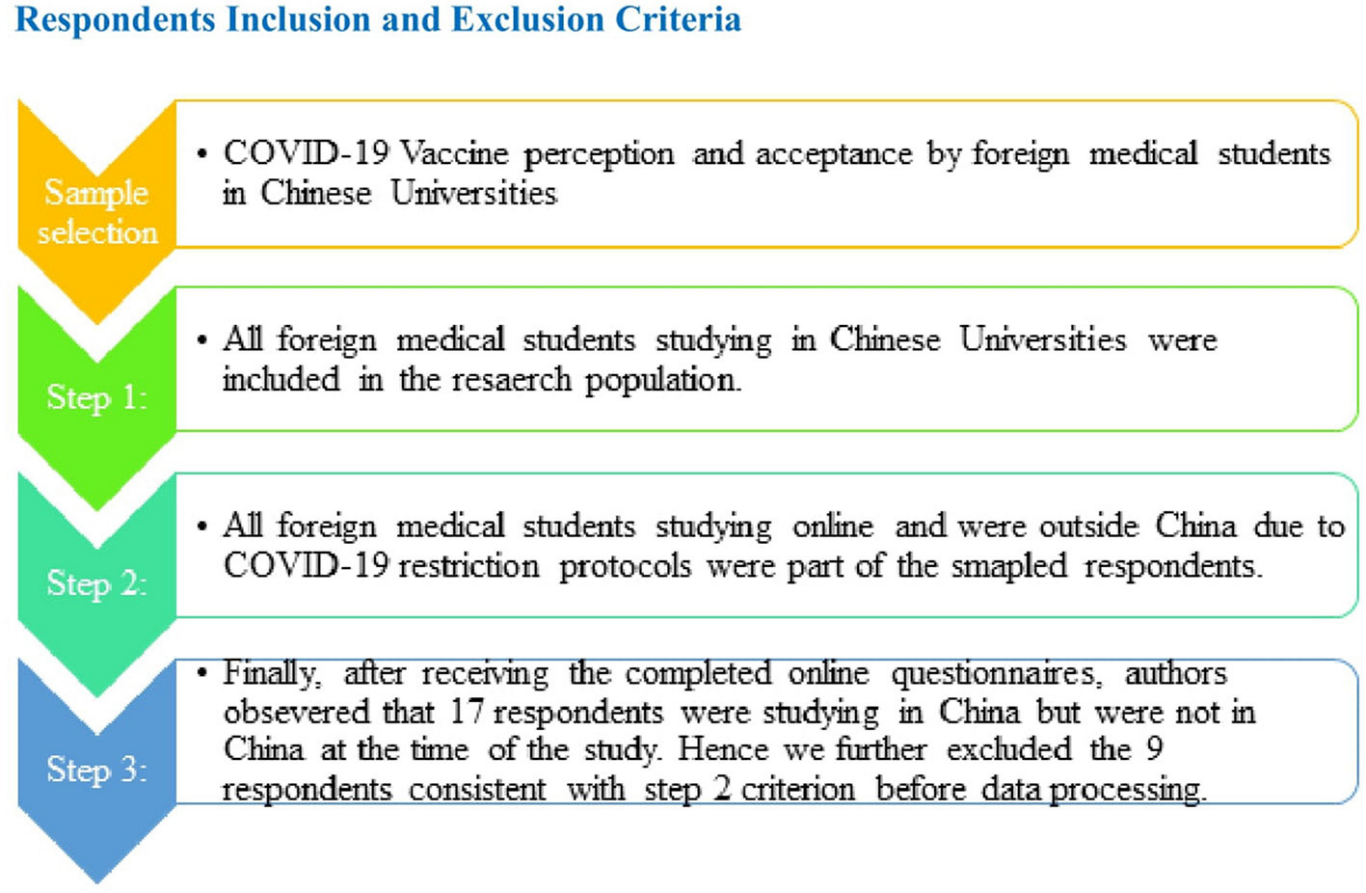
Respondents' inclusion and exclusion flowchart.

As shown in Figure 1, all the medical students studying medical-related majors in China, such as Clinical Medicine and Allied Health Sciences, in any year of study were included as potential respondents. Furthermore, as presented in Figure 1, all the students outside China at the time of the data collection period were not considered. That is, the authors included a respondent caveat (do not fill the questionnaire if you have not been in China since May 2021–April 2022) in the questionnaire. A total of 403 questionnaires were received through the online system. Therefore, to avoid bias and ensure validity in data collection, the authors screened the obtained data and observed that 17 students had completed the questionnaire while they were not in China. So, the authors then excluded 17 respondents, and the sample size remains at 386 for the final data processing.

### Data processing

The obtained data were then processed into categorical and continuous variables. That is, the dependent acceptance is a dummy variable equal to one (1), and non-acceptance or otherwise represents zero (0). The second part of the questionnaire captured data on COVID-19 vaccination acceptability and perceptions formulated based on the literature (18). Also, the authors processed other data into categorical, ordinal, and continuous variables. Finally, in complying with the literature and checking the data internal reliability test in the study, we used the SPSS technique to test the data reliability with Cronbach's alpha which is 0.8. The authors then applied the processed data with multilinear logistic regression techniques.

### Multilinear logistic regression model design

**Figure 2:**
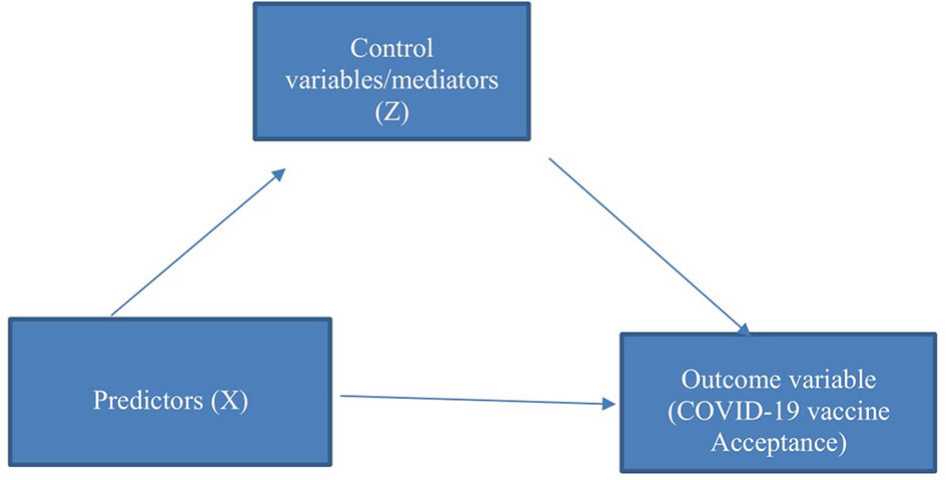
Conceptual framework of COVID-19 vaccine acceptance.

The authors model logistic regression in equations (1–2) to capture the outcome variable, and qualitative term acceptance in a linear relationship with the independent variables (32). The independent variables or predictors are “is COVID-19 vaccine safe in your own opinion”; “how many doses do you think you can receive of COVID-19”; “home continent”; “think that the COVID-19 vaccine is safe with some side effects,” and “think it not possible to get COVID-19 after taking the COVID-19 vaccine.” Also, the authors included control variables (mediator variables) such as gender, age group, marital status, and religion since these may influence the respondents' behavior in different ways. That is, the inclusion of the control variables serves as the mediation channel and this is shown in the conceptual framework (Figure 2).

Furthermore, the authors designed the model, multilinear logistic regression, which is expressed as follows:


(1) $$\begin{array}{l} \ln\left(\frac{p}{1-p}\right)=\;\beta_{0}+\;\beta_{1}x \end{array}$$

(2) $$\begin{array}{l} p=\frac{e\beta_{0}+\beta_{1}x}{1+e\beta_{0}+\beta_{1}x} \end{array}$$

Therefore, we code COVID-19 vaccine acceptance to be equal to one (1). From equation (2), the model can be expressed as follows:


(3) $$\begin{array}{l} p\left(COVID-19\;vaccine\;acceptance=1\right)=\frac{1}{1+e-\left(\beta_{0}+\beta_{1}x+\beta_{2}z\right)} \end{array}$$

Consequently, from equation (3), *p* is the probability, β_0_ is the intercept, β_1_and β_2_ are the parameter coefficients of the predictors and control variables, x as explained above, and z is the control or mediator. The designed model shows the relationship that the probability of accepting the COVID-19 vaccine is related to the independent variables such as the safety of the vaccine, home continent, vaccine side effects, and the number of doses as also explained earlier.

## Results

The authors further followed the STROBE guideline in this section using items 13–17 for presenting the statistical analysis, outcome data, and the main results (34).

### Statistical analysis

**Figure 3:**
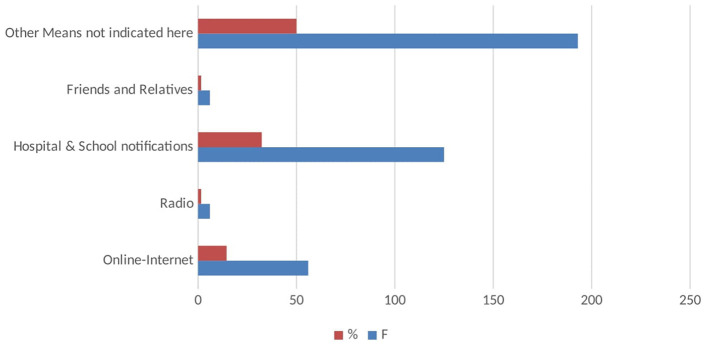
Sources of information regarding COVID-19.

**Figure 4:**
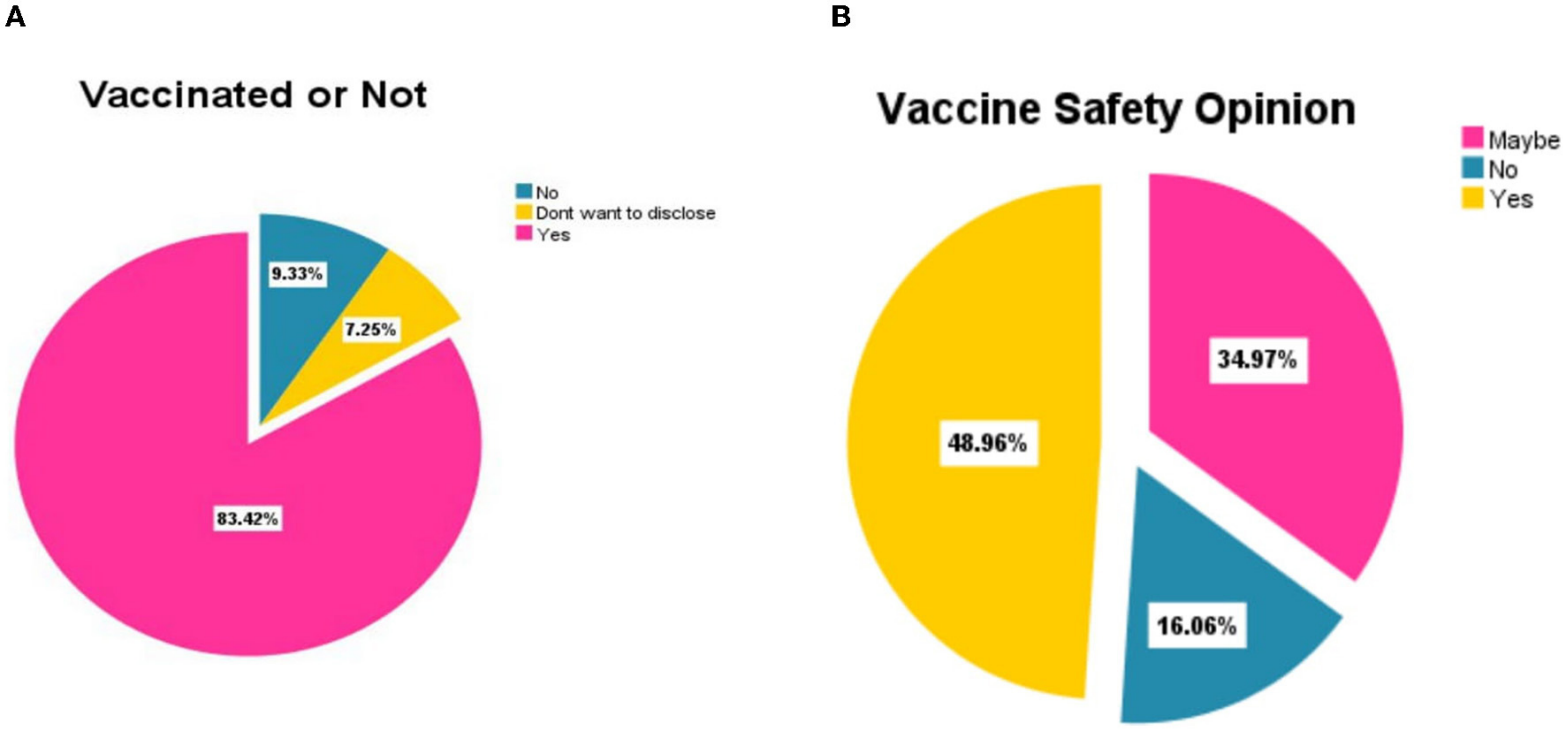
Results of vaccination and vaccine safety opinion of the respondents (*N* = 386). **(A)** Total respondents (*N* = 386) vaccinated or not. **(B)** Total respondents (*N* = 386) on vaccine safety opinion.

The authors used 386 responses after the data processing. Out of the 386 total respondents, 19 (4.9%) were younger than 20 years, 353 (91.5%) respondents were 20–40 years old, and 14 (3.6%) were older than 40 years. In the demographic analysis, there were 140 (36.3%) and 246 (63.7%) female and male students, respectively; please refer to Table 1. In terms of marital status, about 80% were not married because they were undergoing medical training. Furthermore, 239 (61.9%) of the participants were undergraduates, followed by 127 (32.9%) postgraduate and 20 (5.2%) post-doctoral respondents. The demographic statistics additionally show that 56 (14.5%) respondents had accessed COVID-19 vaccine information from the internet, 6 (1.6%) through radios, and 125 (32.4%) from the hospital and school notification prompts. Other means of obtaining COVID-19 information include friends and relatives, with 6 (1.6%) respondents, and 50% using various methods to access COVID-19 vaccine information; detailed information is found in Figure 3. Finally, most of the respondents 88 (22.8%), followed by 74 (19.2%) respondents resided in the North-Eastern and the Central-North regions of China, respectively, see Table 2. The data also reveal that about 324 (83%) respondents indicated that they had been vaccinated. However, about 48.96% agreed it was safe to get the vaccine, and the remaining 51.04% held a contrasting opinion, see Figure 4.

**Table 1:** Social demographic characteristics of the respondents (*N* = 386).

**Variable**	**Category**	***F***	**%**
Gender	Female	140	36.3
	Male	246	63.7
Age-group	less than 20 Years	19	4.9
	Above 20 years less than 40	353	91.5
	Above 40	14	3.6
Marital status	Single	336	87
	Married	50	13
Level of program	Undergraduate	239	61.9
	Postgraduate	127	32.9
	Post Doctorate	20	5.2
Sources of information	Online-Internet	56	14.5
	Radio	6	1.6
	Hospital & School notifications	125	32.4
	Friends and Relatives	6	1.6
	Others	193	50

**Table 2:** Regional location of the participants (*N* = 386).

**Location**	**Region**	***F***	**%**
Region in China	Central North Region	74	19.2
	Central Region	55	14.2
	Central West Region	14	3.6
	Eastern Region	41	10.6
	North West Region	20	5.2
	Northeast Region	88	22.8
	Southern Region	72	18.7
	Western Region	22	5.7
Continent	Africa	281	72.8
	Asia	67	17.4
	Australia	6	1.6
	Europe	12	3.1
	North America	11	2.8
	South America	9	2.3

### Main results and other analysis

#### Multilinear logistic regression results

The estimated model regression analysis revealed the following results. The findings show that six out of the 10 variables are statistically significant (Table 3). The relevant factors which influence the acceptance and perception of the COVID-19 vaccine include the following:

- Gender (β = 0.235, *p* = 0.002) is significant at a 99% confidence level.
- Opinion on the safety of the vaccine (β = 0.081, *p* = 0.043) is significant at 95% confidence.
- Doses of the vaccine to receive (β = 0.175, *p* = 0.001) are significant at a 99% confidence level.
- Vaccine safety with some side effects (β = 0.15, *p* = 0.000) is significant at 99% confidence.
- The possibility of getting coronavirus disease after vaccination (β = 0.062, *p* = 0.040) is significant at a 95% confidence level.
- Home continent (β = −0.062, *p* = 0.071) is significant at a 90% confidence level.

**Table 3:** Factors associated with COVID-19 vaccine acceptance among foreign medical students in China using a multilinear regression model.

**Variables**	**β**	***P***
(Constant)	0.433	0.232
Gender	0.235	0.002^***^
Your age group	−0.026	0.838
Your marital status	0.124	0.280
Study program level	0.022	0.745
Are you affiliated with any religion	0.125	0.227
Your home continent	−0.062	0.071^*^
Is COVID-19 Vaccine safe in your own opinion	0.081	0.043^**^
How many doses do you think you can receive of COVID-19	0.175	0.001^***^
Think that the COVID-19 vaccine is safe with some side effects	0.15	0.000^***^
Think it is not possible to get COVID-19 even after taking the COVID-19 vaccine	0.062	0.040^**^
R^2^	0.86	
N	386	
Durban Watson	1.86

* Significance at 10%;

** Significance at 5%;

*** Significance at 1%.

The table further indicates the regression results obtained from the binary regression model.

Furthermore, based on the results obtained in Table 3, the authors further selected three factors (gender, doses, and home continent) to determine which variable has the greatest influence on acceptance and perception as sensitivity analysis, and the results are presented in Table 4. Table 4 shows that gender is a more significant factor (*p* = 0.000) compared to the number of vaccine doses to be received (*p* = 0.001), with some significance that influences the level of vaccine acceptance among foreign medical students in China compared to the home continent with a significance of p = 0.090. Also, a single (1) vaccine dose was significant compared to triple (3) doses of vaccine based on the reference category (0a) used in Table 1. Moreover, the home continent variable has the least response significance of (0.090) compared to the number of doses (*p* = 0.001). The findings, therefore, suggest that the gender factor has a more powerful influence on vaccine acceptability than the number of doses indicated by the respondents and the home continent factors. The findings hereafter show that various factors influenced the COVID-19 vaccine's acceptance among foreign medical students in China. Also, the results suggest that fear perceptions has affected 200 (51.81%) of the respondent's decision. They feared the vaccines might result in future implications such as infertility, impotence, and systemic health conditions such as cardiovascular, respiratory, or deep vein thrombosis. In addition, 186 (48.19%) respondents also feared that the vaccines were intended to shorten life expectancy.

**Table 4:** Selected variables in logistic regression.

**Variables**	**Category**	**β**	**Sig**.
Threshold	[HOW_LIKELY_SAFE = 1]	−3.199	0.000
	[HOW_LIKELY_SAFE = 2]	−1.599	0.028
Location	[GENDER = 1]	−0.988	0.000^***^
	[GENDER = 2]	0a	.
	[HOME_CONTINENT = 1]	−0.86	0.206
	[HOME_CONTINENT = 2]	0.497	0.503
	[HOME_CONTINENT = 3]	0.419	0.700
	[HOME_CONTINENT = 4]	−0.429	0.623
	[HOME_CONTINENT = 5]	2.116	0.090
	[HOME_CONTINENT = 6]	0a	.
	[DOSES = 1]	−1.115	0.001
	[DOSES = 2]	−0.232	0.370
	[DOSES = 3]	0a	.

^*^Significance at 10%; ^**^Significance at 5%;

*** Significance at 1%.

(1): Reference Category using/based on Table 1.

## Discussion

This section also applied the STROBE checklist, items 18–21 in presenting the key results, discussions, interpretation, generalization, and limitation of the study (34). This study was designed to examine foreign medical students' attitudes, beliefs, experiences, reactions, and feelings in Chinese universities regarding their willingness to receive the COVID-19 vaccine. That is, all the respondents were studying in medical institutions in China. Therefore, the study analysis is relevant to the general public, governments and policymakers, academic scholars, and private institutions in medicine production subject to foreign medical students. The medical students' acceptance and perception of COVID-19 vaccinations may significantly contribute to international students' population behavior on this subject in China. Accordingly, this study analysis may have policy relevance to foreign medical students worldwide on COVID-19 vaccination protocol adoption. Furthermore, this study is an assessment case of foreign medical students in Chinese universities and should apply some caution in its generalization. That is, an extra note must further be taken into consideration in the application of this finding as there may be control factors that influenced the vaccine acceptance that was not included such as government and institutional pressures.

The study finds that the majority of the participants (83.4%) had already received the COVID-19 vaccine at different degrees of doses (some received single, double, or third), and the remaining 6.7% did not yet receive the vaccine at all. These findings are consistent with similar studies in Poland, where it was found that medical students were more than willing to get the vaccine (11, 12). These findings indicate that the impact of medical student's knowledge in their studies would help them have a positive attitude toward the vaccine. First of all, the gender factor in the study discovers that male students have a much higher acceptance level than female students. The finding reveals that the probability of being a male increases the possibility of accepting the COVID-19 vaccine by 23.5% among foreign medical students in Chinese universities, significant at a 99% confidence level. This finding agrees with another study conducted in Yemen, where male students had more level of acceptability and positive perception regarding COVID-19 (13). However, our study finds contrasting results with another study conducted in India which found that female students were more willing than male medical students (12); the differences would be the study design results, country policies' nature, and other external factors. This may be explained by another reason: the majority of male medical students actively participate in various hospital practices.

Furthermore, regarding COVID-19 vaccine safety, the study finds that the majority, about 45.1% of the study participants, had no concerns, while 37.3% had concerns about the vaccine's safety. The participants who recommended the vaccine were more likely and willing to receive the vaccine and 90% less likely to have vaccine hesitancy. Furthermore, the majority of the study participants, about 49%, gave a positive opinion that the vaccine is safe and can prevent COVID-19 disease. In contrast, only 16.1% stated that the vaccine was unsafe and 35% of the participants were not sure about the safety of the vaccine. That could explain why those who recommended the vaccine's safety had enough knowledge about the vaccine and its clinical trials, hence more likely to receive the vaccine than those who disagreed with its safety. The finding reveals that the probability of an increase in vaccine safety leads to more possibility of acceptance of COVID-19 by 8% among foreign medical students in Chinese universities, significant at a 95% confidence level. Our findings agree with another study among health workers, which found that among many factors, COVID-19 risks and safety were the main reasons why the workers took this vaccine (14, 15). The study achieved 49% and gave a positive opinion that the vaccine is safe and can prevent COVID-19 disease, despite being a new study conducted among medical students in China. Furthermore, our findings align with another study conducted elsewhere in India, which established a high positive intention of receiving COVID-19 vaccines (16–18).

In addition, regarding whether to accept or refuse the vaccine among foreign medical students, the study finds that among those who accepted to receive it and those willing to receive it also depends on the opinions of the vaccine's side effects on the human body. The finding reveals that about 37.8% agreed that the vaccine had side effects. Based on the regression estimation results, less probability of side effects would increase the possibility of acceptance of the COVID-19 vaccine by 15% among foreign medical students in Chinese universities, significant at a 99% confidence level. The finding in this study is consistent with a study conducted in the USA, where most people were willing to get vaccinated despite the side effects triggered by external pressure (16, 19). However, about 42.7% of the participants stated that the vaccine was safe without side effects. These findings also support the findings in another current study conducted in Egypt, where they found that many health workers did not get the vaccine because of the fear of side effects and other reasons such as inaccurate information which the media and other sources were using (17, 20). Much as it is found like this, the findings of this study disagree with another study that involved the vaccination of maternity care consumers and providers in Australia, where it was found that the majority of the medical practitioners did not recommend these groups to take vaccination (8, 17, 20). This can result from the fear of side effects for maternity care consumers, which can be associated with.

Easy access to quality information dissemination through radio reveals less response of 1.6%; however, this finding depicts a contrary result from a study that found higher access to information through the radio about COVID-19 (20, 21). The study discovers that current information *via* the internet is systematically becoming a major platform of information for public consumption and whether it is a credible subsequent study might be required to establish it. Being an educational institute affiliated with major hospitals, most (32.4%) students heard about COVID-19 *via* Hospital and school notifications. Such efforts are highly recommendable as they increase the knowledge and awareness of medical-related information (22–27). On the same note, this study also agrees with another recent study conducted in China on international students' safety and COVID-19, which found that the issue of school notices also helped during the combat of COVID-19 in China (21, 22, 28). Vaccination is more effective, even the first shot is good enough to reduce the spread of COVID-19 (25, 28–30). The result shows good acceptance and perception, but other studies had poor knowledge and positive perception among those respondents (26, 27, 30–33). Our study depicted that based on the adequate information provided to the students, it boosts confidence, ensuring convenience and reduced complacency for vaccine acceptance and hesitancy, and this is consistent with the WHO-SAGE 3-Cs model (32, 34).

Furthermore, the findings show that 72.8% of the respondents are from Africa, and the rest, 27.2% are from Asia, Australia, Europe, North America, and South America. The estimated regression statistic on the parameter home continent shows a negative impact on COVID-19 vaccine acceptance (-0.062) and is significant at a 90% confidence level. This suggests that people on different continents or countries may resist accepting the COVID-19 vaccine and this is consistent with (31, 34–36). That is, the probability increase of people in Africa (as the reference) continents among medical students reduce the acceptance of the COVID-19 vaccine by more than 6% in Chinese universities, and this is significant at a 90% confidence level. This may be due to language and information barriers. These results are supported by the data statistic of respondents (6.7%) who still need to receive the vaccine. This finding confirms the literature and, however, the hesitancy rate of 6% among foreign medical students in China is lesser compared to 10% (31), 30% (30) in India, and 37% in Uganda (32).

## Limitations of the study

First and foremost, due to the COVID-19 lockdown protocols particularly on the Chinese University campuses, the authors applied the online (survey and questionnaire) data collection technique. Therefore, this study is not without responses and information biases, and difficulty in interpreting the respondent sentiments behind some answers received. However, it was the most effective means considering some lockdowns where the researchers could not go. Also, it is worth noting that the assessment of vaccination intention in this study did not account for other relevant factors influencing vaccination and the respondent's decisions, such as vaccine length of protection and the requirement for booster doses, which could impact participants' decisions. That is, the study did not also control for external factors (such as government and institutional pressure) on medical students, which may have some bias in the study.

## Conclusion

This study examined foreign medical students' attitudes, beliefs, experiences, reactions, and feelings regarding the COVID-19 vaccine acceptance and perception. We found that the opinion on the vaccine's safety, doses of the vaccine to receive, vaccine safety with some side effects, and the possibility of getting coronavirus disease after vaccination are significant positive factors influencing the acceptability in China among foreign medical students. Also, the finding further showed that foreign medical students from different continents adversely affect COVID-19 vaccine acceptance and perception among foreign medical students. The implication is that vaccine hesitancy among medical students is primarily affected by continent factors. The study, therefore, concluded that the COVID-19 vaccine is crucial, mainly influenced by the continent consideration in which the medical students are originally from. This, therefore, has policy implications for government and public health administration. Even though there is evidence of COVID-19 vaccine hesitancy among foreign medical students, the rate is reasonably low (6%) compared to other countries such as India and Uganda. We conclude that there is 83% of COVID-19 acceptance among foreign medical students in Chinese universities. This may be mainly due to the safety and effectiveness of health systems, knowledge, and vaccine information availability.

## Data availability statement

The original contributions presented in the study are included in the article/Supplementary material, further inquiries can be directed to the corresponding author.

## Ethics statement

The studies involving human participants were reviewed and approved by Ethics Committee of First Hospital of Shanxi Medical University. The patients/participants provided their written informed consent to participate in this study.

## Author contributions

CA: took part in drafting, revising, critically reviewing the article, and acquisition of data. ZD: study design and took part in drafting. HA: formal analysis, revising, modeling, and critically reviewing the article. ML: acquisition of data. GM: formal analysis, modeling and drafting, and revising. CZ: ethical consideration process and acquisition of data. SZ: analysis. RH: acquisition of data and ethical consideration process. SO: visualization and review. AD: acquisition of data. LJ: execution. DZ: interpretation. HZ: revising technical support. HH: conception and critically reviewing the article. All authors gave final approval of the version to be published, have agreed on the journal to which the article has been submitted, and agreed to be accountable for all aspects of the work.

## Footnotes

^1^Foreign medical students in China: https://www.china-admissions.com/study-medicine-in-china/.
